# Case Report: All That Glisters Is Not^*^ Cancer

**DOI:** 10.3389/fmed.2020.541629

**Published:** 2020-11-30

**Authors:** Claudio Tirelli, Chandra Bortolotto, Patrizia Morbini, Giulia Maria Stella

**Affiliations:** ^1^Unit of Respiratory System Diseases, Department of Medical Sciences and Infectious Diseases, Istituto di Ricovero e Cura a Carattere Scientifico (IRCCS) Policlinico San Matteo Foundation, Pavia, Italy; ^2^Unit of Radiology, Department of Intensive Medicine, Istituto di Ricovero e Cura a Carattere Scientifico (IRCCS) Policlinico San Matteo Foundation and University of Pavia Medical School, Pavia, Italy; ^3^Unit of Pathology, Department of Molecular Medicine, Istituto di Ricovero e Cura a Carattere Scientifico (IRCCS) Policlinico San Matteo Foundation and University of Pavia Medical School, Pavia, Italy

**Keywords:** lung cancer, sarcoidosis, metastases, staging, origin

## Abstract

Properly performed staging in non-small-cell lung cancer (NSCLC) is necessary to avoid wrong therapeutic decisions. Here we present a case which manifested as advanced NSCLC but ultimately was composed of two different and rare pathologies. The first is a TTF-1 positive axillary lymph node that could be defined either as an unusual isolated differentiated cancer of unknown primary or as an even rarer case of ectopic lung epithelium which underwent malignant transformation. The second is sarcoidosis, a sarcoid-like alteration, in remission after oral steroids. The main implication of a correct diagnosis regards patient outcome and the avoidance of toxic inappropriate systemic chemotherapy.

## Introduction

Lung cancer is a major cause of death in both men and women worldwide. It is frequently associated to distant metastasis, most often discovered at the time of diagnosis; thus, correct disease staging, including molecular profile analysis, is fundamental in order to properly subtype cancer and for the subsequent choice of the most effective treatment.

## Case Report

**Figure 1:**
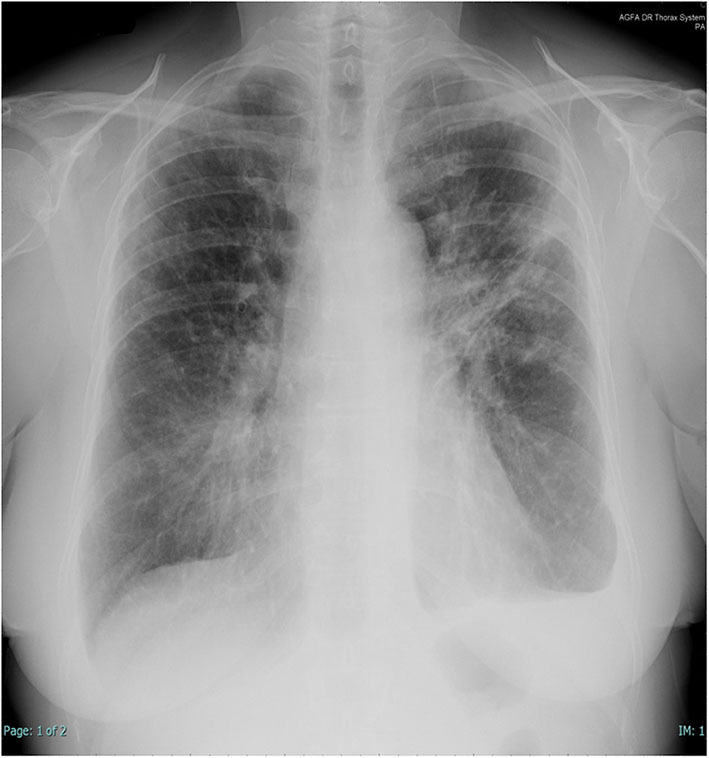
Chest X-rays at presentation. Left subclavian pulmonary thickening associated with patchy bilateral peripheral opacity particularly represented in the left lung where there are also band-like consolidations and pleural effusion.

**Figure 2:**
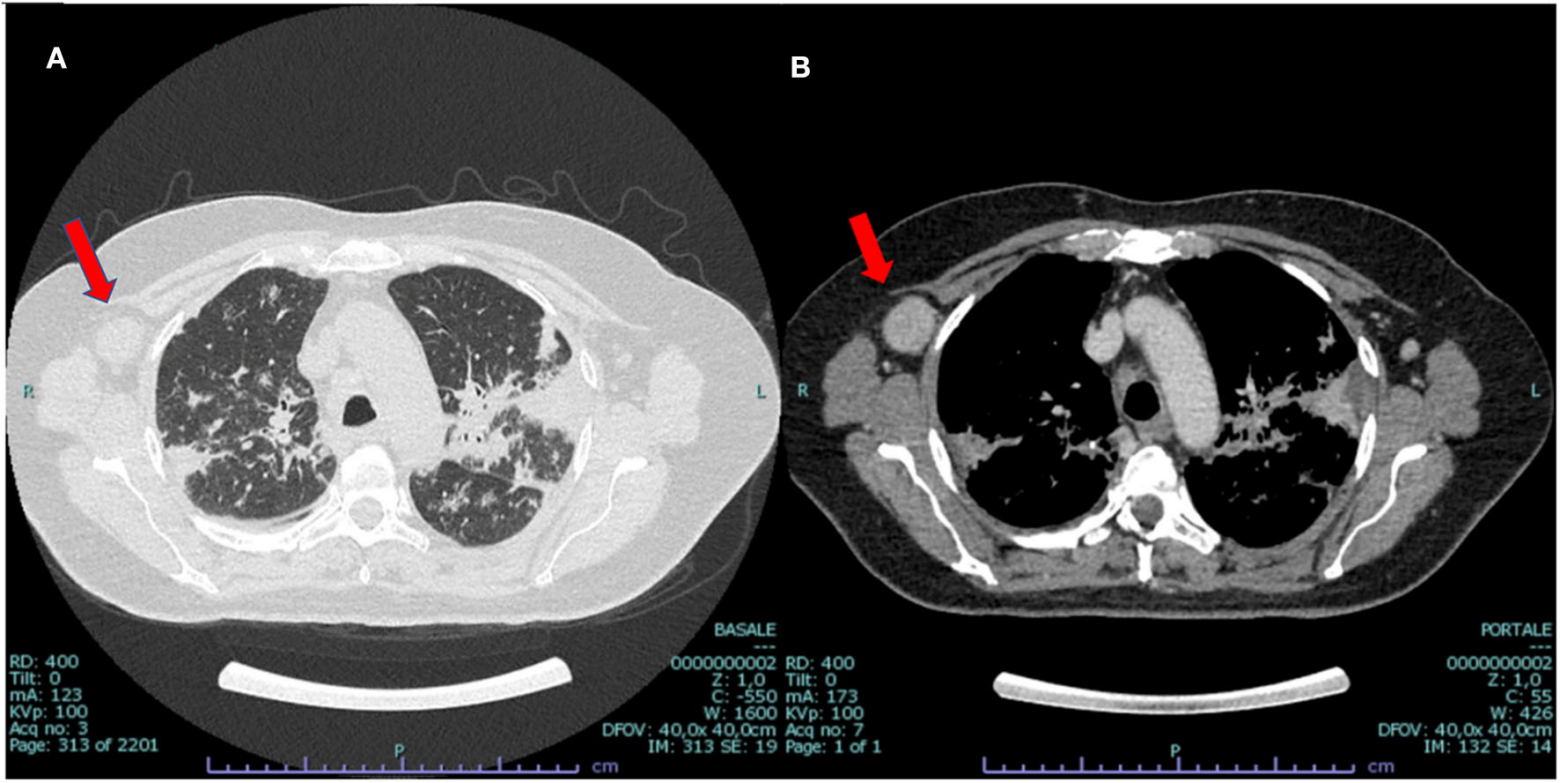
CT scan at presentation: bilateral parenchymal infiltrates **(A)** and mediastinal hilar lymph node enlargement **(B)**, suggestive of sarcoidosis but also compatible with malignant origin. Enlarged vascularized pathologic (3 cm) axillar node detected in the right axillary region (red arrow).

**Figure 3:**
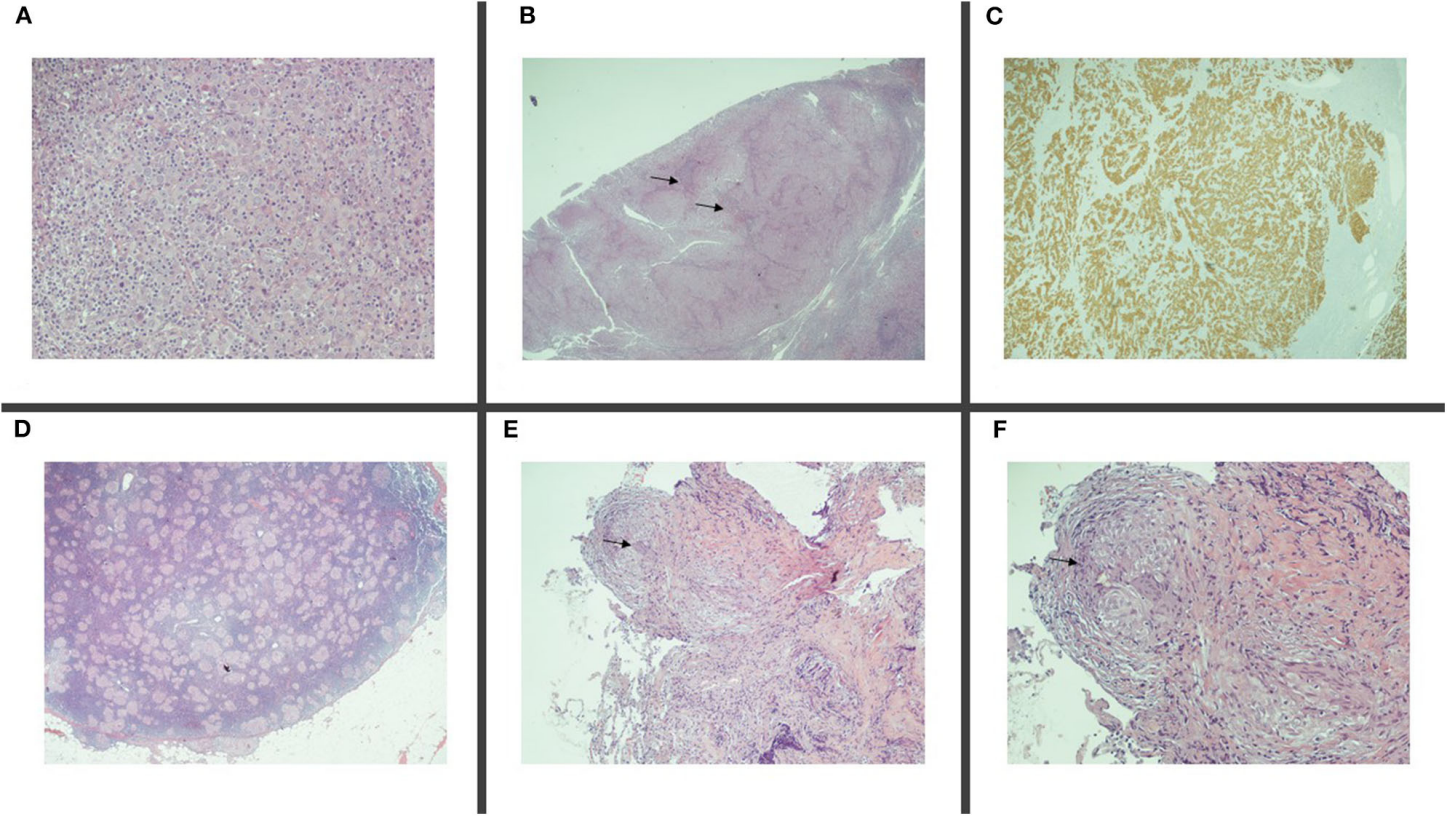
Histologic samples. Surgical sample of lymph node localization of adenocarcinoma, ×20 **(A)**, ×5 (**B**, → ), featuring positive staining for cytokeratin 7 **(C)**, and concomitant epithelioid sarcoid-like granuloma, ×5 **(D)**. Transbroronchial biopsy ×10 **(E)** and ×20 **(F)** displaying sarcoid non-necrotizing epithelioid granulomas (→ ).

**Figure 4:**
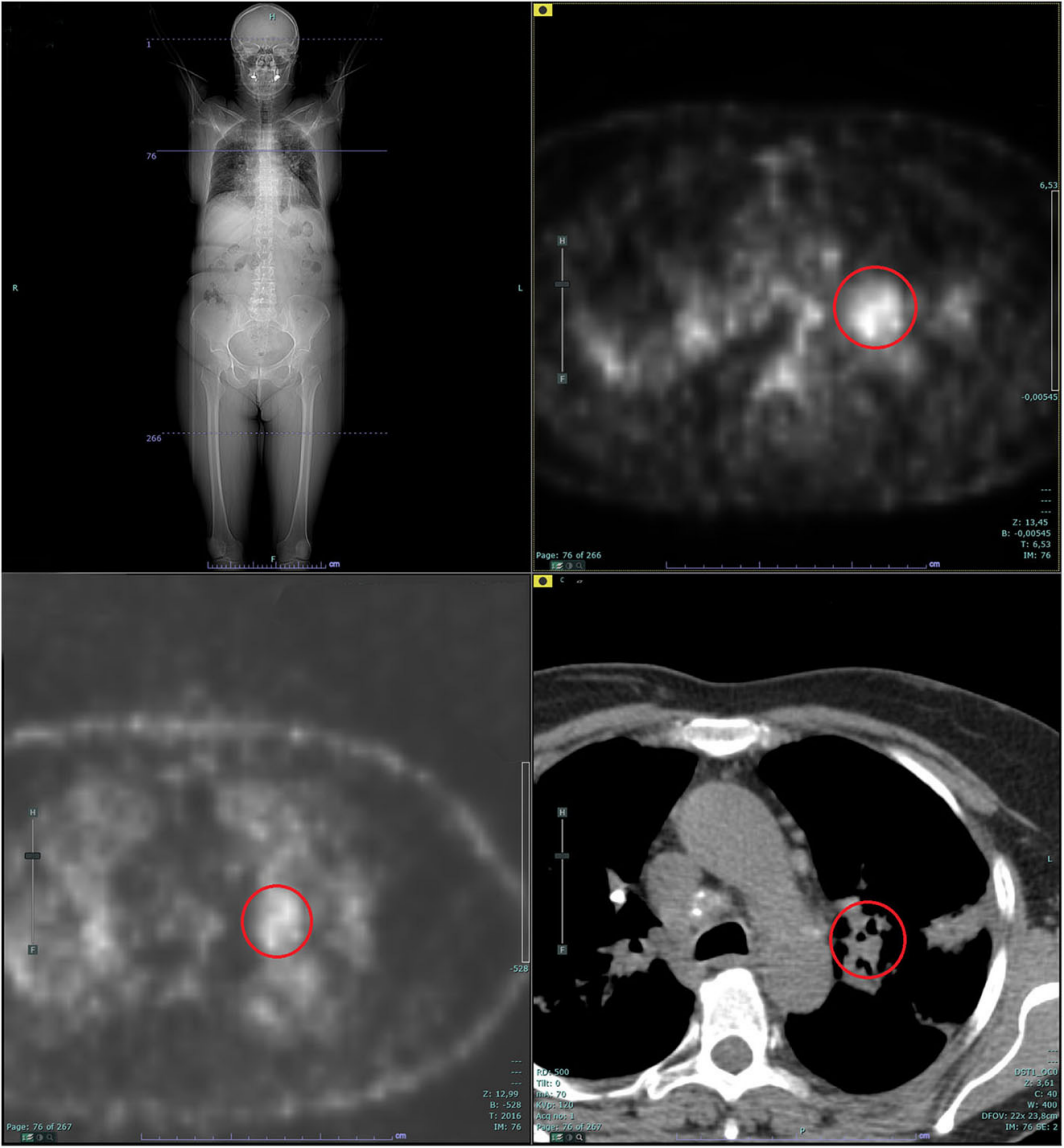
Whole-body positron emission tomography (PET): supra- and subdiaphragmatic pathologic lymphadenopathies in the absence of putative primary mass detection. The red circle shows the pathologic standardized uptake value (SUV) at left hilar lymph node (L10 station).

**Figure 5:**
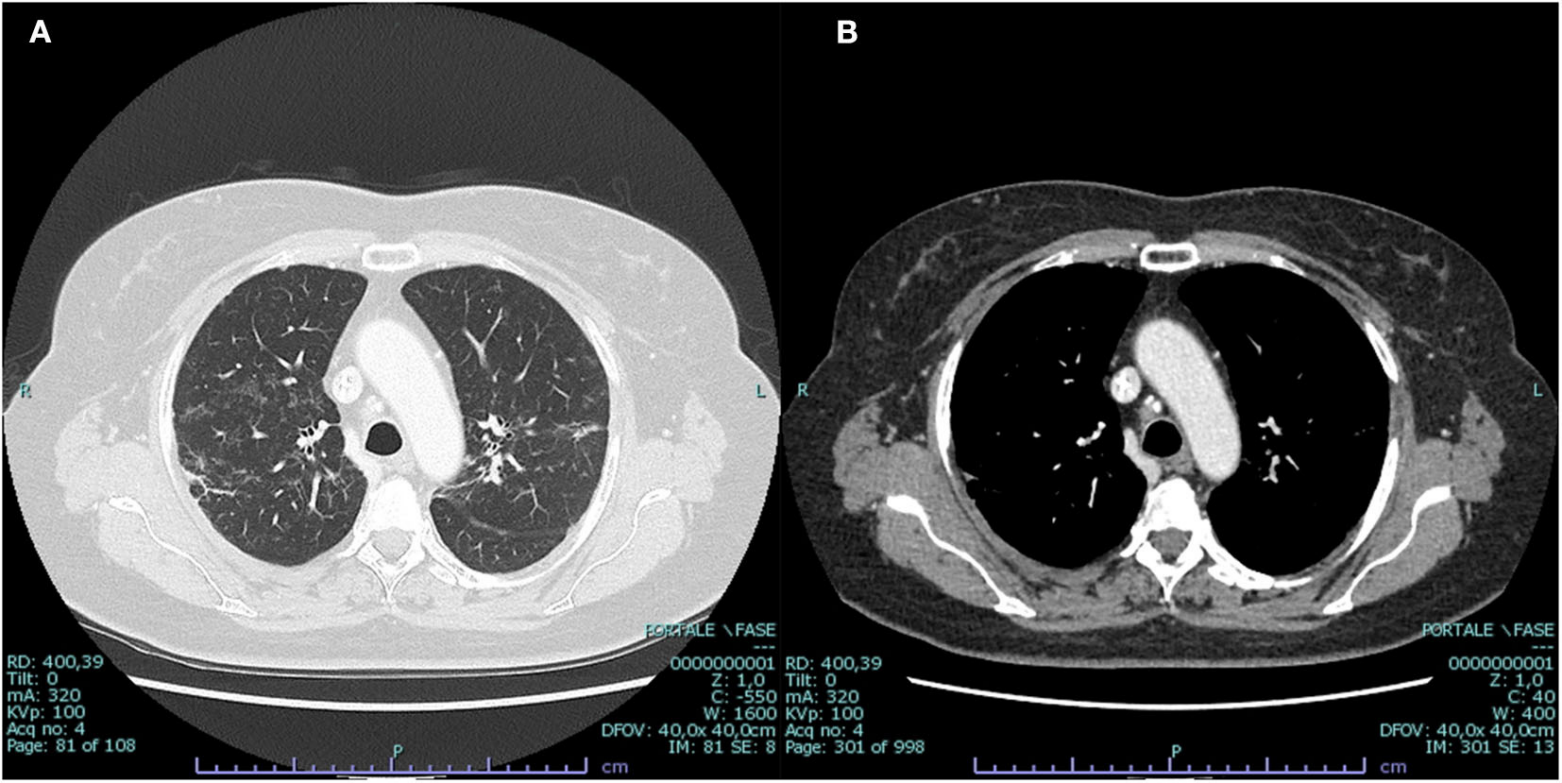
CT scan after treatment: remaining parenchymal fibrosis in upper lobes compatible with sarcoidosis grade IV **(A)**, without mediastinal or extra-thoracic lymph node enlargement **(B)**.

**Figure 6:**
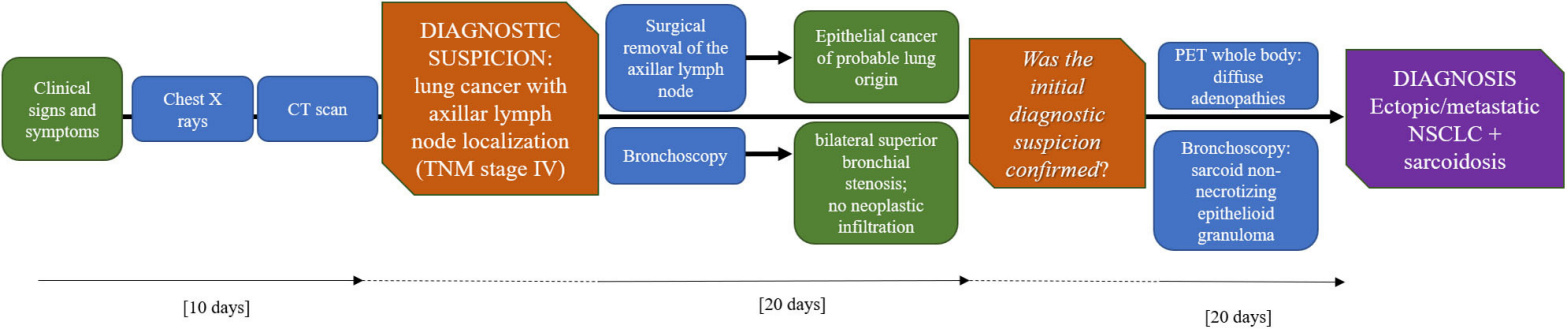
Timeline with the most relevant data of the clinical case.

In 2014, a 60-year-old non-smoker female with past medical history of recurrent bronchitis came to visit for 3 months of dry cough and exertional dyspnea, which remained unchanged after treatment with clarithromycin. At chest examination, bilateral diffuse crackles could be appreciated, without wheezing. No touchable lymphadenopathies were present. Echocardiogram was normal, while on chest X-ray (CXR; Figure 1) parenchymal infiltrates were documented, which were more evident in the left lung. A total body CT scan (TB CT; Figure 2) revealed diffuse parenchymal infiltrates with mediastinal and hilar lymphadenopathies, suggestive of sarcoidosis but also compatible with a malignant origin. A vascularized pathologic (3 cm) lymph node was also detected in the right axillary region and surgically removed 8 days after the CXR and 2 days after the CT scan, together with other nodes of the same station, for diagnostic purposes. At histologic examination, localization/metastasis of poorly differentiated epithelial neoplasia was found in only one of the resected nodes (Figures 3A–C), whereas in the others giant cell granulomatous epithelioid sarcoid-like reaction was detected (Figure 3D). An exhaustive and multidisciplinary diagnostic workup was thus initiated to determine the putative primary origin of cancer cells. Their immunophenotype (cytokeratin 7+, AE1/AE3+, TTF1+, and p63-) was coherent with axillary lymph node localization of lung adenocarcinoma (1, 2). In detail, the cytokeratin panel expression confirmed the epithelial origin, while the positivity of thyroid transcription factor 1 (TTF-1) was a highly specific marker for lung as primary origin and suggested the adenocarcinomatous lineage of differentiation. The absence of p63 expression allowed to exclude squamous differentiation as well as neuroendocrine carcinomas. Subsequent molecular analysis documented the absence of actionable genetic targets since mutational analysis revealed *EGFR* wt, *KRAS* wt, *ALK* not translocated, and *HER2/neu* not amplified. We then proceeded with a first bronchoscopy to study deeper the lung parenchyma and find the primary site of malignant growth. A transbronchial biopsy (TBB) was then performed, revealing bilateral superior bronchial stenosis in the absence of histologic neoplastic infiltration (Figures 3E,F). To complete the disease stage and/or eventually to detect a distant site of origin of the neoplastic cells, a whole-body positron emission tomography was performed and evidenced supra- and subdiaphragmatic pathologic lymphadenopathies in the absence of putative primary mass detection (Figure 4). Since no tumor masses were demonstrated by imaging, a second bronchoscopy with TBB was thus repeated to deeply analyze the lung parenchyma. Quite unsuspectedly, the TBB obtained specimens that were free from neoplastic cells but rich of sarcoid non-necrotizing epithelioid granulomas. Steroid treatment (prednisone, 1 mg/kg) was started, with rapid improvement on symptomatology. After 1 month, a TB CT showed reduction in parenchymal infiltrates and lymphadenopathies without extra-thoracic neoplastic or sarcoid localizations. Being in the presence of carcinoma featuring lung epithelial lineage of origin vs metastatic lung cancer without detectable primary mass, in the absence of the expression of actionable targets, platinum-based conventional chemotherapy (cisplatin-pemetrexed) was started, and steroids were tapered until 10 mg/day. The TB CT at the end of three cycles of adjuvant chemotherapy confirmed the presence of some fibrotic branches, compatible with stage IV pulmonary sarcoidosis, without oncologic localizations (Figure 5). A timeline with all relevant data from this clinical case is available in Figure 6. The steroid regimen was gradually reduced to a maintenance of 5 mg/day over a period of 1 year. At 5 years from diagnosis, the result at follow-up is still negative, with no neoplasm recurrence and with good control of sarcoidosis in the absence of steroid treatment.

## Comments

We report a case of coexistent ectopic/metastatic lung cancer (in the absence of detectable primary mass in the lung parenchyma) and sarcoidosis. The latter most often occurs in younger people, but it can be present at any age. Females are overrepresented in the age group 50–60 years, and the disease is then often chronic and already advanced at presentation (3), as in the present case. The relationship between cancer and sarcoidosis is still controversial (4, 5), although a moderate but significantly increased cancer risk in patients with sarcoidosis has been reported (6). On this basis, sarcoidosis has been proposed as a risk factor for lung cancer in never-smokers, but no conclusive data are available until now (7). Three hypotheses have been proposed regarding the relationship between cancer and sarcoidosis: (1) sarcocentric: sarcoidosis develops before lung cancer and could induce neoplastic transformation through cell-mediated immune abnormalities in the absence of activation of known oncogenic drivers, (2) oncocentric: sarcoidosis might represent an immunological reaction to disperse cancer antigens, and (3) the two entities might be independent. The mechanistic explanation of the pathogenesis of cancer associated with sarcoid-like granulomas and/or sarcoidosis is still unknown. A quite recent hypothesis suggested that molecular mimicry could occur because of the altered activity of oncogenes and ultimately lead to crossed-type mediated body reactions targeting structurally similar sections or regions from the tissue homeostasis (8). Interestingly, it should be noted that the occurrence of sarcoidosis-like granulomas is involved also in response to novel cancer therapies since they might represent important immune checkpoint inhibitor-related reactions (9).

Notably, the patient far surpassed the expected survival for a stage IV non-small-cell lung cancer (NSCLC). This point allows us to conclude that malignant transformation of ectopic lung epithelium was more probable than metastatic lung disease; thus, surgical removal was, in that case, radical. However, it cannot be excluded that a small primary lung mass was initially present and that it subsequently evolved in dormancy or regression. Indeed the process of metastatic dissemination begins when malignant cells start to migrate and leave the primary mass (10). The latter define a rare but non-negligible disease (over 300,000 new cases per year worldwide) which is called cancer of unknown primary (2). The disease shares common traits: (i) early metastatic dissemination, (ii) unpredictable metastatic organ distribution, (iii) lack of organ- or tissue-specific differentiation, and (iv) extremely aggressive potential and poor prognosis in case of disseminated disease. The present case is worth to be described since it is paradigmatic of how adequate staging in NSCLC is mandatory to avoid wrong therapeutic decisions. What seemed to be consistent with a stage IV lung cancer requiring quite obvious therapeutic approach was instead composed of two different pathologies. The first is a TTF-1 positive axillary lymph node that could be defined either as an unusual isolated differentiated metastasis without a sure primary site of origin or as an even rarer case of ectopic lung epithelium which underwent malignant transformation. It was successfully removed in the absence of neoplastic recurrence. The second is sarcoidosis or, a sarcoid-like alteration, remaining after surgery but in persistent remission with a cycle of oral steroids. Given the improvement/reversibility and the presence of lung parenchymal and lymph node involvement and based also on Scadding staging on X-rays, there is a possibility that this was stage II sarcoidosis (11, 12). Overall, this quite unexpected diagnosis was allowed by invasive diagnostic procedures on both the hypothetical nodal metastasis and the primary site of the disease. The main implication of this approach regards patient prognosis and therapeutic management by avoiding the toxicity of an inappropriate treatment for advanced cancer.

## Ethics Statement

Written informed consent was obtained from the individual(s) for the publication of any potentially identifiable images or data included in this article.

## Author Contributions

CT was responsible for data collection and wrote the paper. CB and PM collected data. GS supervised and wrote the paper. All authors contributed to the article and approved the submitted version.

## Conflict of Interest

The authors declare that the research was conducted in the absence of any commercial or financial relationships that could be construed as a potential conflict of interest.
